# Efficacy of the combination use of aprepitant and palonosetron for improving nausea in various moderately emetogenic chemotherapy regimens

**DOI:** 10.1186/s40360-018-0278-2

**Published:** 2019-01-14

**Authors:** Naohisa Yoshida, Tetsuya Taguchi, Masayoshi Nakanishi, Ken Inoue, Tetsuya Okayama, Takeshi Ishikawa, Eigo Otsuji, Koichi Takayama, Haruo Kuroboshi, Motohiro Kanazawa, Yoshito Itoh

**Affiliations:** 1 Department of Molecular Gastroenterology and Hepatology, Graduate School of Medical Science, Kyoto Prefectural University of Medicine, 465 Kajii-cho, Kawaramachi-Hirokoji, Kamigyo-ku, Kyoto, 602-8566 Japan; 2 Outpatient Cancer Chemotherapy Center, Kyoto Prefectural University of Medicine Hospital, Kyoto, Japan; 3 Division of Endocrinological and Breast Surgery, Kyoto Prefectural University of Medicine, Kyoto, Japan; 4 Division of Digestive Surgery, Graduate School of Medical Science, Kyoto Prefectural University of Medicine, Kyoto, Japan; 5 Department of Pulmonary Medicine, Kyoto Prefectural University of Medicine, Kyoto, Japan; 6 Department of Obstetrics and Gynecology, Kyoto Prefectural University of Medicine, Kyoto, Japan; 7 Department of Urology, Graduate School of Medicine, Kyoto Prefectural University of Medicine, Kyoto, Japan

**Keywords:** Palonosetron, Aprepitant, MEC, Nausea, Chemotherapy

## Abstract

**Background:**

Nausea is more difficult to control than vomiting in chemotherapy. We therefore analyzed the efficacy of a strong supportive treatment with aprepitant, palonosetron, and dexamethasone against nausea for various moderately emetogenic chemotherapy (MEC).

**Methods:**

A total of 312 cases treated by palonosetron with or without aprepitant receiving MEC regimens using oxaliplatin, carboplatin, and irinotecan from 2014 to 2016 in our outpatient center for digestive organ cancers, lung cancers, and gynecological cancers were analyzed. Through propensity score matching analysis, cases were divided into 97 cases receiving 2 drugs (palonosetron+dexamethasone) and 97 receiving 3 drugs (aprepitant+palonosetron+dexamethasone). We examined the control rates of nausea for the first two consecutive courses in the both groups. Additionally, risk factors for acute and delayed nausea were analyzed using a multivariate analysis among overall 312 cases.

**Results:**

The control rates of nausea in the two- and the three-drug groups were as follows: acute, 92.8 and 95.9% (*p* = 0.35); and delayed, 83.5 and 81.4% (*p* = 0.85), although the control rates of vomiting exceeded 95% in both groups. A multivariate analysis showed that significant risk factors for acute nausea (odds ratio, 95% confidence interval) were elevation of serum creatinine (12.601, 2.437–65.157), general fatigue (3.728, 1.098–12.661), and performance status (PS) 2 (19.829, 3.200–122.865). The significant risk factors for delayed nausea were elevation of alanine aminotransferase (2.397, 1.153–4.984), general fatigue (2.652, 1.380–5.097), and PS 2 (5.748, 1.392–23.740).

**Conclusions:**

The control for nausea in MEC was insufficient even with palonosetron and aprepitant, and we should pay attention to risk factors for preventing nausea.

## Background

Chemotherapy-induced nausea and vomiting (CINV) is a major side effect that can reduce the oral intake and adversely affect the patient’s quality of life [1]. Nausea in particular is reported to have a stronger negative impact on patients’ daily lives than vomiting [2]. Indeed, a study showed that the food intake decreased by 300–500 kcal/day even in patients with mild nausea compared to patients with no nausea [3]. Therefore, it is very important to prevent all degrees of nausea in order to maintain a good patient status during chemotherapy.

Recent guidelines for antiemetic treatments, such as the National Comprehensive Cancer Network (NCCN), Multinational Association of Supportive Care in Cancer (MASCC), the American Society of Clinical Oncology (ASCO), and the Japanese guidelines, describe the classifications of emetogenic risks for each anticancer drug and regimen [4–7], resulting in categories of high emetogenic chemotherapy (HEC), moderate emetogenic chemotherapy (MEC), low emetogenic, and minimal emetogenic chemotherapy. Among MEC regimens, two-drug treatments, such as the first-generation 5-hydroxytryptamine-3 receptor antagonist (5 HT3RA) and dexamethasone (DEX), are regularly recommended, and additional aprepitant is needed for some drugs, such as carboplatin (CBDCA) and irinotecan (CPT-11). Aprepitant is a neurokinin 1 receptor antagonist (NK1RA), and its effectiveness against delayed CINV has been reported in many studies on HEC and MEC regimens [8, 9]. However, a study of an oxaliplatin (L-OHP)-based regimen for colorectal cancer showed that the control rate of acute and delayed nausea (mild to severe) by 3-drugs therapy (NK1RA+ 5HT3RA + DEX) was 93.6 and 66.3%, respectively, compared to 2-drugs therapy (5HT3RA + DEX) (acute: 90.2%, *p* = 0.23, delayed: 61.8%, *p* = 0.36) though first-generation 5HT3RA was used in almost half of cases [9].

Palonosetron is a second-generation 5HT3RA with a longer half-life than the first-generation 5HT3RA [10]. According to several guidelines, three-drug treatment including palonosetron is recommended for preventing CINV due to HEC [4–7]. On the other hand, the ASCO and MASCC guidelines recommend that palonosetron should be used for MEC regimens when NK1RA is not used [5, 6]. A review of palonosetron showed the control rates for acute and delayed nausea in palonosetron+DEX in various MEC regimens to be 70–80% and 60-%70%, respectively, which were better than those of the first-generation 5HT3RA was still low [10]. Thus, nausea is not sufficiently prevented according to the recommendations of the recent guidelines.

In this study, we hypothesized that the strongest three-drug treatment (NK1RA + panlonosetron+DEX) may show better control for delayed nausea for MEC regimens than regular recommended treatments and analyzed the efficacies of strong three-drug treatments for nausea associated with various MEC regimens.

## Methods

**Fig. 1:**
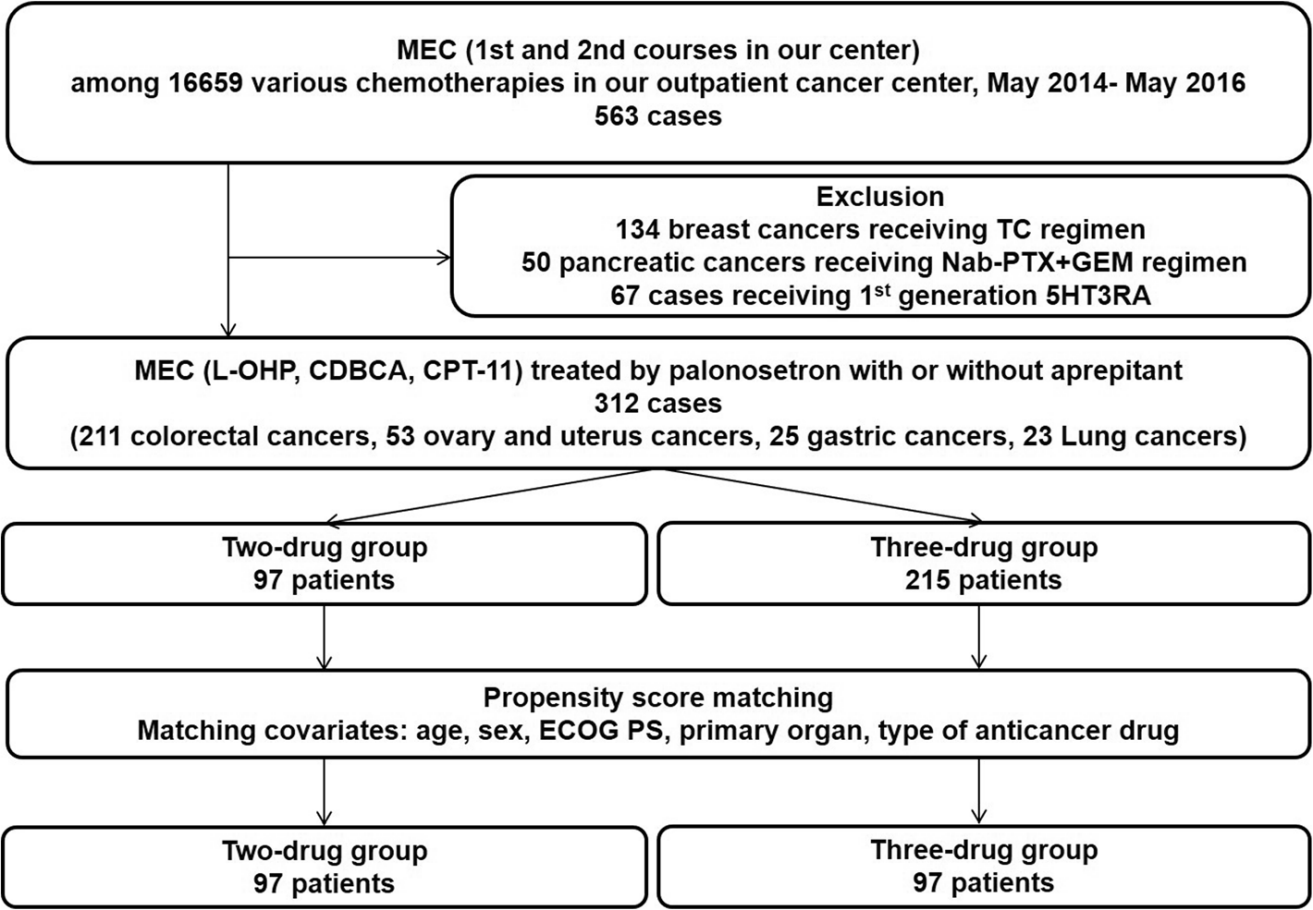
Study flow

This was an observational study. Among 16,659 patients having various cancers from May 2014 to May 2016 in our outpatient cancer chemotherapy center, 563 cases receiving MEC was analyzed. To analyze MEC regimens using L-OHP, CDBCA, and CPT-11, 134 patients with breast cancer and patients with 50 pancreatic cancer were excluded. Then, the cumulative 312 cases treated by palonosetron in MEC with or without NK1RA using L-OHP, CBDCA, and CPT-11 for digestive organ cancers (colorectal cancer and gastric cancer), lung cancers, and gynecological cancers (ovarian cancer, cervical cancer, endometrial cancer) were analyzed (Fig. 1).

According to anti-emetic therapies, the 312 cases were divided into 97 cases receiving 2 drugs (palonosetron and DEX) and 215 cases receiving 3 drugs (NK1RA, palonosetron, and DEX). Then, we performed a propensity score matching to control and to reduce selection bias in each group. Matching covariates were as follows: age, sex, Eastern Cooperative Oncology Group (ECOG) scale performance status (PS) scale, primary organ, and type of anticancer drug. Finally, 97 cases in the two-drug group and 97 cases in the three-drug group were analyzed (Fig. 1). The first-course chemotherapy and its associated CINV were analyzed in all patients. However, some patients received first-course chemotherapy at admission; for those cases, the second-course chemotherapy and its associated CINV were analyzed. In patients receiving multiple courses of MEC, the data for two consecutive courses of chemotherapy in our outpatient center were analyzed. Additionally, which 5HT3RA such as first-generation or palonosetron were administered in both the two- and three-drug groups, were decided by each doctor according to patient characteristics including those who received CINV as a previous chemotherapy. In addition, whether or not NK1RA was used was also decided by each doctor. The exclusion criteria were as follows: patients with severe organ disorders; patients having complications that induced nausea and/or emesis (e.g. brain metastases and ulcerative diseases); and the administration of drugs that cause nausea and/or emesis during the investigation period, except for antiemetics (e.g. major or minor tranquilizers, or corticosteroids for any other reason).

In both the two- and three-drug groups, various clinical characteristics, including the age, sex, ECOG PS scale, primary organ, type of anticancer drug, sequence of chemotherapy (first course or not-first course), and additional oral DEX day 2–3 days of chemotherapy were analyzed [11]. The control rates of nausea and vomiting were analyzed in both groups along with the rates for acute phase (< 24 h) and delayed phase (≥24 h). Additionally, the control rates for nausea and vomiting for each anticancer drug (L-OHP, CBDCA, CPT-11, and oral 5-fluorouracil (5-FU)) were also examined in both groups. Furthermore, all 312 cases before propensity score matching were divided into cases with or without nausea and various clinical factors, such as patient characteristics (age, sex, PS) and symptoms (pain, constipation, diarrhea, oral mucositis, neuropathy, general fatigue) and blood examination findings (neutrophil value [NEUT], hemoglobin [Hb], platelet [PLT], creatinine [CRE], alanine aminotransferase [AST], aspartate aminotransferase [ALT]). A multivariate analysis was performed to clarify the risk factors of acute and delayed nausea in all 312 cases receiving palonosetron.

In this study, all symptoms including nausea and vomiting were recorded using a questionnaire, which was used in our center since 2012 for all patients. The questionnaire was written by each patient in our center with a help of our qualified nurses who didn’t know this study. The questionnaire was made according to the National Cancer Institute – Common Toxicity Criteria (NCI-CTC) grade ver. 4.0 after every course of chemotherapy [12]. Regarding pain, it was scored using a numerical rating score (NRS: 0–10).

This study was approved by the ethics committee of Kyoto Prefectural University of Medicine and was carried out in accordance with the World Medical Association Helsinki Declaration (adopted in 1964 and amended in 1975, 1983, 1989, 1996, 2000, 2002, 2004, and 2008).

### Chemotherapy regimens

Regarding colorectal cancer, patients on mFOLFOX6 received concurrent L-OHP (85 mg/m^2^) and leucovorin (LV) (400 mg/m^2^) for 2 h on day 1 followed by bolus 5-FU (400 mg/m^2^) and subsequent continuous infusion of 5-FU (2400 mg/m^2^) over 46 h [13]. Patients on SOX or XELOX received oral S-1 (80–120 mg/body) or oral capecitabine (2000 mg/m^2^/day) twice daily after breakfast and dinner from days 1 to 14 and intravenous L-OHP (130 mg/m^2^) for 2 h on day 1 repeated every 3 weeks [14, 15]. Concomitant intravenous bevacizumab (7.5 mg/kg) or cetuximab (400 mg/m^2^ for the first administration, 250 mg/m^2^ for the second and subsequent administrations) was administered before L-OHP. FOLFIRI (l-LV 200 mg/m^2^, day 1; CPT-11150 mg/m^2^, day 1; 5-FU bolus 400 mg/day, day 1; 5-FU ci 2400 mg/m^2^, 48 h; cetuximab every 2 weeks), IRIS (S-1: 80–120 mg/body, day 1–14; CPT-11125 mg/m^2^, day 1 and 15, every 4 weeks), and CPT-11 (CPT-11: 150 mg/m^2^, day 1 and 15, every 4 weeks) were administered according to the standard protocol outlined in the Japanese guideline [13, 16].

Regarding these regimens, cetuximab, bevacizumab, or panitumumab were added appropriately for each patient. Other regimens for various cancers were described as follows: In brief, for gynecological cancers, TC (paclitaxel (PTX): 180 mg/m^2^, day 1; CDBCA: AUC5, day 1, every 3 weeks) and DC (docetaxel (DTX): 60 mg/m^2^, day 1; CBDCA: AUC5, day 1, every 3 weeks) were administered [17]. For lung cancers, PTX + CBDCA (PTX: 200 mg/m^2^, day 1, CBDCA: AUC6, day 1, every 3 weeks) and etoposide (ETP) + CBDCA (ETP: 80 mg/m^2^, day 1; CBDCA: AUC5, day 1, every 3 weeks) were administered [18].

Among all chemotherapy, whether additional oral DEX (4–8 mg) on day 2–3 days of chemotherapy was administered in both the two- and three-drug groups, were decided by each doctor according to patient characteristics.

### Statistical analyses

Statistical analyses were performed using the Mann-Whitney *U* test and chi-square test. Continuous variables, such as the patient age, were analyzed using the Mann-Whitney *U* test. A propensity score-matching analysis between the two groups was performed to reduce the effect of any selection bias including sex, age, primary organ, and so on. The propensity score-matching method was proposed to evaluate statistically causal effects free from confounding effects by mathematically refashioning an observational study into a randomized study [19, 20]. Propensity scores were estimated using a logistic regression model with various covariates described above. By using these propensity scores, patients were individually matched between the two groups (a 1:1 matching). Multivariate logistic regression analyses were also performed to determine the risk factors of acute and delayed nausea. In detail, only factors showing *P* < 0.05 in the univariate analysis were examined. A *p*-value < 0.05 was considered to be statistically significant. All statistical analyses were performed using using SPSS version 22.0 (IBM Corp., Armonk, NY, USA).

## Results

The patients characteristics in the two-drug and three-drug groups before and after propensity score matching are shown in Table 1. After matching, there were no significant differences in the age (66.4 ± 9.9 vs. 64.7 ± 9.5, *p* = 0.49), or ratio of male sex (46.4 vs. 38.1, *p* = 0.24), between the two-drug group and three-drug group, nor in the ECOG PS. Additionally, there were no significant difference in the primary organ and type of anticancer drugs between the two groups. In addition, regarding the sequence of chemotherapy, the respective ratios for 1st and 2nd-3rd courses were 40.2 and 59.8% in the two-drug group and 46.4 and 53.6% in the three-drug group (*p* = 0.38).

**Table 1:** Patient characteristics in the two-drug (palonosetron+DEX) and three-drug (NK1RA + palonosetron+DEX) groups before and after a propensity score-matching

	Two-drug before matching	Three-drug before matching	*p* value	Two-drug after matching	Three-drug after matching	*p* value
Case number	97	215		97	97	
Age (mean ± SD)	66.4 ± 9.9	63.2 ± 11.2	0.011	66.4 ± 9.9	64.7 ± 9.5	0.49
Sex (M/F), n (%)	45/52	103/112	0.80	45/52	37/60	0.24
	(46.4/53.6)	(47.9/52.1)		(46.4/53.6)	(38.1/61.9)	
ECOG PS0/1,2, n (%)	54/43	96/119	0.07	54/43	51/46	0.67
	(55.7/44.3)	(44.7/55.3)		(55.7/44.3)	(51.5/48.5)	
Primary organ, n (%) Colon/Ovary+Uterus/Lung/Stomach	56/16/14/11	155/37/9/14	0.003	56/16/14/11	57/24/7/9	0.24
	(57.7/16.5/14.4/11.3)	(72.1/17.2/4.2/6.5)		(57.7/16.5/14.4/11.3)	(58.8/24.7/7.2/9.3)	
Type of anticancer drugs, n (%) L-OHP/CBDCA/CPT-11	46/28/23	132/46/37	0.07	46/28/23	52/31/14	0.26
	(47.4/28.9/23.7)	(61.4/21.4/17.2)		(47.4/28.9/23.7)	(53.6/33.0/14.4)	
Sequence of chemotherapy, n (%) 1st course/2nd-3rd courses	39/58	88/127	0.90	39/58	45/52	0.38
	(40.2/59.8)	(40.9/59.1)		(40.2/59.8)	(46.4/53.6)	
Additional oral DEX on day 2–3 of chemotherapy, *n* (%)	9 (9.3)	30 (14.0)	0.25	9 (9.3)	12 (12.4)	0.49

*NK1RA* neurokinin 1 receptor antagonist, *DEX* dexamethasone, *M* male, *F* female, *PS* performance status, *L-OHP* oxaliplatin, *CBDCA* carboplatin, *CPT-11* irinotecan, *SD* standard deviation

**Fig. 2:**
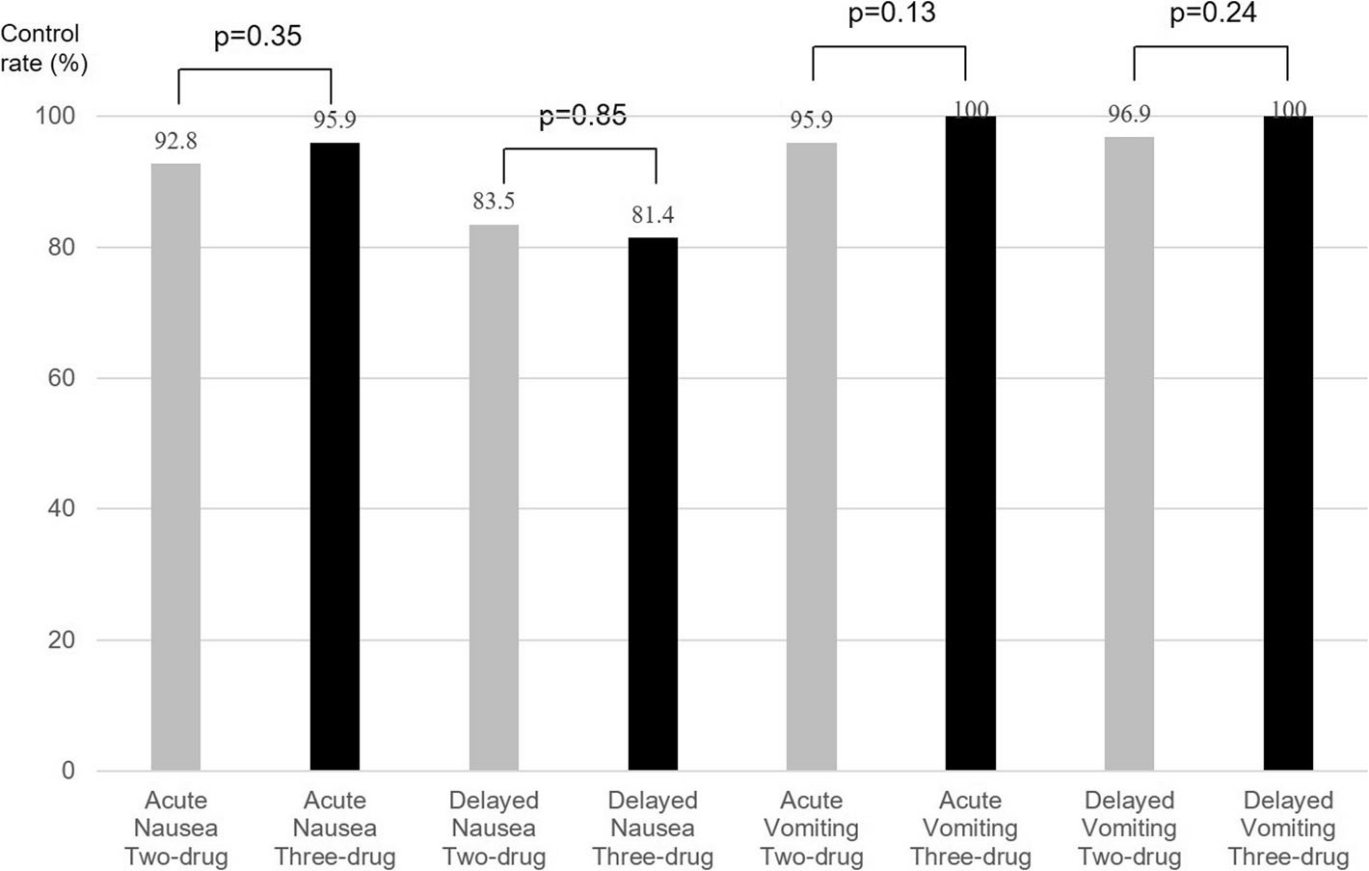
The control rates for acute and delayed nausea and vomiting in the two-drug (palonosetron+DEX) and three-drug (NK1RA + palonosetron+DEX) groups after a propensity score matching

The control rates of acute nausea in the two- and three-drug groups were 92.8 and 95.9% (*p* = 0.35), respectively, and those of delayed nausea were 83.5 and 81.4% (*p* = 0.85), respectively (Fig. 2). The control rates of acute vomiting in the two- and three-drug groups were 95.9 and 100.0% (*p* = 0.13), respectively, and those of delayed nausea were 96.9 and 100.0% (*p* = 0.24), respectively.

According to each anti-cancer drug, the control rates for nausea in the two- and three-drug groups were 82.6 and 76.9% (*p* = 0.49) for L-OHP, respectively, 78.6 and 80.6% (*p* = 0.84) for CBDCA, respectively, 91.3 and 100.0% (*p* = 0.70) for CPT-11, respectively, and 84.2 and 75.0% (*p* = 0.30) for oral 5-FU, respectively. The control rates for vomiting in the two- and three-drug groups were 95.7 and 100.0% (*p* = 0.42) for L-OHP, respectively, 96.4 and 100.0% (*p* = 0.95) for CBDCA, respectively, 91.3 and 100.0% (p = 0.70) for CPT-11, respectively, and 94.7 and 100.0% (*p* = 0.37) for oral 5-FU, respectively.

The results of an analysis of the cases with or without overall nausea and among all 312 cases administered palonosetron are shown in Table 2. There were significant differences in the median age (60.4 ± 12.6 vs. 65.2 ± 10.2 years), *p* < 0.001), constipation (42.4% vs. 31.8%, *p* = 0.03), neuropathy (71.2% vs. 53.6%, p = 0.03), and general fatigue (74.2% vs. 44.3%, p < 0.001) between the cases with and without overall nausea, respectively.

**Table 2:** Results of the analysis of cases with or without overall nausea among 312 cases receiving palonosetron

	Cases with nausea	Cases without nausea	*p* value
Case number	66 (21.2%)	246 (78.8%)	
Age, mean ± SD	60.4 ± 12.6	65.2 ± 10.2	< 0.001
≥75 years, n (%)	5 (7.6)	42 (17.1)	0.05
≥70 years, n (%)	19 (28.8)	91 (37.0)	0.21
Sex, n (%)	28:38	120:126	0.35
	(42.4:57.6)	(48.8:51.2)	
PS 0:1 + 2, n (%)	26:40	129:117	0.05
	(39.4:60.6)	(52.4:47.6)	
Aprepitant, n (%)	50 (75.8)	165 (67.0)	0.17
Neut < 1000, n (%)	5 (7.6)	18 (7.3)	0.63
Hb < 10 mg/dl, n (%)	32 (48.5)	142 (57.7)	0.18
CRE ≥1.1 mg/dl, n (%)	7 (6,5)	7 (2.8)	0.07
ALT > 2.5 ULN, n (%)	17 (25.8)	38 (15.9)	0.05
Constipation, n (%)	28 (42.4)	70 (31.8)	0.03
Diarrhea, n (%)	19 (28.8)	48 (19.0)	0.10
Oral mucositis, n (%)	20 (30.3)	57 (20.8)	0.23
Neuropathy, n (%)	47 (71.2)	139 (53.6)	0.03
General fatigue, n (%)	49 (74.2)	113 (44.3)	< 0.001
Pain NRS ≥1, n (%)	22 (33.3)	69 (31.5)	0.40
L-OHP, n (%)	42 (63.6)	136 (55.3)	0.22
CPT-11, n (%)	8 (12.1)	52 (21.1)	0.09
CBDCA, n (%)	16 (24.2)	58 (23.6)	0.91
Oral intake of 5-FU, n (%)	39 (59.1)	116 (47.2)	0.08

*PS* performance status, *NEUT* neutrophil, *ULN* upper limit of normal, *NRS* numerical rating score, *L-OHP* oxaliplatin, *CPT-11* irinotecan, *CBDCA* carboplatin, *SD* standard deviation

The results of univariate and multivariate analyses of the risk factors for acute nausea are shown in Table 3. The multivariate analysis showed the significant risk factors to be elevation of CRE (odds ratio [OR], 12.601; 95% confidence interval [CI], 2.437–65.157; *p* = 0.003), general fatigue (OR, 3.728; 95%CI, 1.098–12.661, *p* = 0.035), and PS 2 (OR, 19.829; 95% CI, 3.200–122.865; *p* = 0.001).

**Table 3:** Results of univariate and multivariate analyses of risk factors of acute nausea among 312 cases with palonosetron receiving MEC

	Univariate						Multivariate				
	*n*	OR	95% CI			*P*-value	OR	95% CI			*P*-value
Age < 75 vs. ≥75 years	312	n.c.					–				
Sex Female (vs. Male)	312	1.162	0.510	,	2.647	0.720	–				
Anti-cancer drug	312						–				
L-OHP	178	1.000		ref							
CPT11	60	1.676	0.636	,	4.420	0.296					
CBDCA	74	0.920	0.316	,	2.679	0.878					
Oral 5-FU	312						–				
Nothing	157	1.000		ref							
S-1	30	1.859	0.556	,	6.210	0.314					
Capecitabine	125	0.938	0.382	,	2.301	0.888					
Additional oral steroid days 2 or 3 (vs. Nothing)	312	1.203	0.531	,	2.726	0.658	–				
NK1RA (vs. Nothing)	312	1.175	0.474	,	2.913	0.728	–				
Primary organ	312						–				
colorectal cancer	211	1.000		ref							
gastric cancer	25	n.c.									
lung cancer	23	1.516	0.412	,	5.572	0.531					
gynecological cancer	53	0.606	0.173	,	2.131	0.435					
Hb < 10 mg/dl	312	1.010	0.443	,	2.301	0.981	–				
Neut < 1500/μl	312	0.528	0.068	,	4.096	0.541	–				
PLT < 7.5 × 10^4^/μl	312	1.078	0.473	,	2.457	0.858	–				
CRE ≥1.1 mg/dl	312	6.643	1.848	,	23.881	0.004	12.601	2.437	,	65.157	0.003
AST > 2.5 ULN	312	1.576	0.651	,	3.815	0.313	–				
ALT > 2.5 ULN	312	1.936	0.767	,	4.890	0.162	–				
Fever elevation > 38 °C	312	1.696	0.363	,	7.921	0.502	–				
Constipation	312	1.218	0.506	,	2.933	0.661	–				
Diarrhea	312	3.241	1.397	,	7.522	0.006	2.594	0.984	,	6.834	0.054
Oral mucositis	312	2.189	0.940	,	5.099	0.069	–				
Taste alteration	312	1.895	0.833	,	4.314	0.128	–				
Neuropathy	312	2.916	1.064	,	7.986	0.037	1.088	0.346	,	3.423	0.885
Eruption	312	3.630	1.470	,	8.965	0.005	2.012	0.716	,	5.657	0.185
General fatigue	312	4.085	1.492	,	11.180	0.006	3.728	1.098	,	12.661	0.035
NRS ≥1	312	1.406	0.598	,	3.309	0.435	–				
PS	312										
0	155	1.000		ref			1.000		ref		
1	146	3.435	1.215	,	9.708	0.020	2.568	0.820	,	8.043	0.105
2	11	25.000	5.668	,	110.271	0.000	19.829	3.200	,	122.865	0.001

*MEC* moderately emetogenic chemotherapy, *L-OHP* oxaliplatin, *CPT-11* irinotecan, *CBDCA* carboplatin, *Hb* hemoglobin, *Neut* neutrophil, *PLT* platelet, *CRE* creatine, *ULN* upper limit of normal, *NRS* numerical rating scale, *PS* performance status, *OR* odds ratio, *CI* confidence interval

The results of univariate and multivariate analyses of the risk factors for delayed nausea are shown in Table 4. The multivariate analysis showed the significant risk factors to be elevation of ALT (OR, 2.397; 95% CI, 1.153–4.984; *p* = 0.019), general fatigue (OR, 2.652; 95% CI, 1.380–5.097, p = 0.003), and PS 2 (OR, 5.748; 95% CI, 1.392–23.740; *p* = 0.016).

**Table 4:** Results of univariate and multivariate analyses of risk factors of delayed nausea among 312 cases with palonosetron receiving MEC

	Univariate						Multivariate				
	*n*	OR	95% CI			*P*-value	OR	95% CI			*P*-value
Age < 75 vs. ≥75 years	312	0.444	0.168	,	1.176	0.102	–				
Sex Female (vs. Male)	312	1.273	0.724	,	2.239	0.402	–				
Anti-cancer drug	312										
L-OHP	178	1.000		ref			1.000		ref		
CPT11	60	0.396	0.159	,	0.989	0.047	0.505	0.137	,	1.860	0.304
CBDCA	74	0.983	0.509	,	1.898	0.960	1.769	0.533	,	5.870	0.351
Oral 5-FU	312										
Nothing	157	1.000		ref			1.000		ref		
S-1	30	0.812	0.261	,	2.530	0.720	0.671	0.144	,	3.124	0.611
Capecitabine	125	1.817	1.011	,	3.266	0.046	2.111	0.690	,	6.458	0.190
Additional oral steroid days 2 or 3 (vs. Nothing)	312	0.654	0.370	,	1.156	0.144	–				
NK1RA (vs. Nothing)	312	1.340	0.715	,	2.513	0.362	–				
Primary organ	312						–				
colorectal cancer	211	1.000		ref							
gastric cancer	25	n.c.									
lung cancer	23	1.054	0.371	,	2.998	0.921					
gynecological cancer	53	1.111	0.539	,	2.291	0.776					
Hb < 10 mg/dl	312	0.719	0.410	,	1.260	0.249	–				
Neut < 1500/μl	312	0.632	0.181	,	2.207	0.472	–				
PLT < 7.5 × 10^4^/μl	312	1.535	0.875	,	2.691	0.135	–				
CRE ≥1.1 mg/dl	312	3.112	0.953	,	10.167	0.060	–				
AST > 2.5 ULN	312	1.780	0.964	,	3.287	0.066	–				
ALT > 2.5 ULN	312	2.166	1.122	,	4.180	0.021	2.397	1.153	,	4.984	0.019
Fever elevation > 38 °C	312	0.574	0.127	,	2.595	0.471	–				
Constipation	312	0.884	0.470	,	1.664	0.702	–				
Diarrhea	312	1.394	0.729	,	2.666	0.315	–				
Oral mucositis	312	1.230	0.655	,	2.310	0.520	–				
Taste alteration	312	1.225	0.685	,	2.190	0.493	–				
Neuropathy	312	1.804	0.986	,	3.302	0.056	–				
Eruption	312	1.534	0.875	,	2.690	0.135	–				
General fatigue	312	3.221	1.729	,	6.000	0.000	2.652	1.380	,	5.097	0.003
NRS ≥1	312	1.125	0.613	,	2.064	0.704	–				
PS	312										
0	155	1.000		ref			1.000		ref		
1	146	1.532	0.853	,	2.752	0.153	1.360	0.723	,	2.558	0.340
2	11	4.549	1.285	,	16.101	0.019	5.748	1.392	,	23.740	0.016

*MEC* moderately emetogenic chemotherapy, *L-OHP* oxaliplatin, *CPT-11* irinotecan, *CBDCA* carboplatin, *Hb* hemoglobin, *Neut* neutrophil, *PLT* platelet, *CRE* creatine, *ULN* upper limit of normal, *NRS* numerical rating scale, *PS* performance status, *OR* odds ratio, *CI* confidence interval

## Discussion

Previous reports on HEC regimens have shown that the control rate of 3-drug treatment using NK1RA + first-generation 5HT3RA + DEX for vomiting (51–73%) is significantly better than those of two-drug treatments, such as first-generation 5HT3RA + DEX (42–52%) [21–24]. However, with respect to nausea, the control rates are only 33–49% even with three-drug treatments and 24–44% with two-drug treatments (not significant). However, a study (TRIPLE trial) on HEC including cisplatin (> 50 mg/m^2^) showed that NK1RA + palonosetron+DEX controlled delayed nausea and vomiting better than NK1RA + first-generation 5HT3RA + DEX (67.0% vs. 59.0%, *p* = 0.0142) [25], Another study of three-drug treatment with palonosetron for HEC including cisplatin (> 50 mg/m2) showed no nausea rates were 61.5–70.4% during 6 cycles of chemotherapy [26]. These results suggest that palonosetron might have higher efficacy against delayed nausea than first-generation 5HT3RA. A RCT of MEC regimens using L-OHP also showed that the control rates for nausea in the acute and delayed phases were better (93.4 and 83.2%) in the two-drug combination (palonosetron+DEX) than those (82.7 and 70.9%, *p* < 0.001, *p* = 0.005) in the three-drug combination (NK1RA + first-generation 5HT3RA + DEX) [9]. Our rates (92.8 and 83.5%) for palonosetron+DEX were also similar to these previously reported findings. Furthermore, our study failed to show the superiority of control for acute and delayed nausea with NK1RA + palonosetron+DEX (95.9 and 81.4%, respectively) compared to these rates with palonosetron+DEX. This finding suggests that two-drug treatment (palonosetron+DEX) is sufficient for achieving control of nausea in MEC regimens, although the control rate was not enough. This two-drug treatment is also recommended due to good compliance without a prescription for NK1RA as well as its low cost compared with three drug treatment (NK1RA + palonosetron+DEX).

However, a small Japanese crossover study of 35 patients receiving MEC regimens using L-OHP and CPT-11 showed the total control rate for severe to mild nausea to be 46% for palonosetron+DEX and 60% for NK1RA + first-generation 5HT3RA + DEX (*p* = 0.235) [27]. This result is not consistent with the result of our study. Thus, a large prospective randomized study should be performed to prove which treatments (e.g. palonosetron+DEX or NK1RA + first-generation 5HT3RA + DEX) are better for controlling nausea associated with various MEC regimens.

Regarding vomiting, a study comparing two-drug (palonosetron+DEX) and three-drug combinations (NK1RA + first-generation 5HT3RA + DEX) for an L-OHP-based regimen showed the rates of no vomiting in the acute and delayed phases to be 99.4 and 95.5%, respectively, with the 2-drug combination [28]. Our rates of no vomiting in the acute and delayed phases in the two-drug group (palonosetron+DEX) were 95.9 and 96.9%, respectively, which were almost the same as in that previous study. In addition, our data showed the rates of no vomiting in the acute and delayed phases in the three-drug group (NK1RA + palonosetron+DEX) to be 100.0 and 100.0%, respectively. This suggests that two-drug treatment (palonosetron+DEX) was sufficient to control vomiting associated with an MEC regimen. Indeed, ASCO and MASCC/ESMO recommend palonosetron for MEC when NK1RA is not used [4, 5].

Even though MEC regimens include various kinds of anti-cancer drugs, anti-emetic treatments for MEC are largely decided by guidelines [4–6]. We analyzed regimens with L-OHP, CBDCA, and CPT-11, which are all defined as MEC regimens. Regarding vomiting, palonosetron controlled more than 90% of cases in patients receiving each anti-cancer drug, regardless of inclusion in the 2- or 3-drug group. However, regarding nausea, the control rates ranged widely (78.6–91.3%) in the 2-drug group and were not markedly improved in the 3-drug group (75.0–100.0%). Furthermore, the control rates with CBDCA and L-OHP were lower than that with CPT-11 both in both groups. Previous studies have shown the control rate of vomiting and nausea in the CBDCA regimen to be 89.5 and 60.5% in the acute and delayed phase, respectively, with a 3-drug regimen of NK1RA + palonosetron+DEX [29]. Another study of MEC regimens using L-OHP and CPT-11 showed the overall control rate to be 69.1% and 66.7% with a 2-drug regimen of palonosetron+DEX [30]. Thus, physicians must be alert for nausea in patients receiving regimens of CBDCA and L-OHP. As we showed in the present study, it didn’t improve even in three drug treatment including NK1RA. Thus, additional anti-emetic drugs, such as 5HT2 blockers, gastrointestinal motility activator such as metoclopramide, and olanzapine should be considered [4–7, 31]. These drugs are more economical than NK1RA. We also suggest that MEC regimens be divided into subgroups based on their rates of emesis to ensure better supportive care.

Risk factors typically associated with CINV include female gender, a younger age, a poor PS, no drinking of alcohol, and no smoking [32–39]. However, most of these risk factors were assessed concerning their association with vomiting and severe to moderate nausea. In the present study, we clarified the risk factors of all grades (severe to mild) of acute and delayed nausea. Elevation of CRE, general fatigue, and PS 2 were extracted as risk factors for acute nausea. In addition, elevation of ALT, general fatigue, and PS 2 were extracted as risk factors for delayed nausea. For patients with these risk factors, additional anti-emetic drugs should be considered.

To our knowledge, this is the first report to describe elevation of CRE and ALT as risk factors for CINV. We should therefore pay close attention to patients with those factors. In such patients, the addition of anti-emetic agents, such as 5H2 blockers and olanzapine, to palonosetron+DEX may be considered [4–7]. We further suggest that additional NK1RA is not necessary for controlling nausea in patients receiving palonosetron+DEX, as there were no significant differences in the control rates for nausea between NK1RA1 + palonosetron+DEX and palonosetron+DEX.

### Study limitations

This study was a retrospective review of patient medical records. Two- and three-drug treatment were decided by each doctor according to each patient’s characteristics. Thus, there was a possibility of selection bias concerning inclusion in the two- and three-drug groups though we performed a propensity score matching.

## Conclusions

The control rates of nausea with NK1RA + palonosetron+DEX in MEC using L-OHP, CPT-11, and CBDCA were not sufficient, especially for delayed nausea. In addition, the rates were not superior to those with palonosetron+DEX. Elevation of ALT, general fatigue, and PS 2 were considered risk factors for delayed nausea. We should therefore pay particularly close attention to patients with these risk factors.

## Abbreviations

5-HT3RA: 5-hydroxytryptamine-3 receptor antagonist
5-FU: 5-fluorouracil
ALT: Aspartate aminotransferase
ASCO: The American Society of Clinical Oncology
AST: Alanine aminotransferase
CBDCA: Carboplatin
CI: Confidence interval
CINV: Chemotherapy-induced nausea and vomiting
CPT-11: Irinotecan
CRE: Creatine
DEX: Dexamethasone
DTX: Docetaxel
ECOG: Eastern Cooperative Oncology Group
ETP: Etoposide
Hb: Hemoglobin
HEC: High emetogenic chemotherapy
L-OHP: Oxaliplatin
LV: Leucovorin
MASCC: Multinational Association of Supportive Care in Cancer
MEC: Moderate emetogenic chemotherapy
NCCN: National Comprehensive Cancer Network
NCI-CTC: National Cancer Institute – Common Toxicity Criteria
NEUT: Neutrophil value
NK1RA: A neurokinin 1 receptor antagonist
NRS: Numerical rating score
OR: Odds ratio
PLT: Platelet
PS: Performance status
PTX: Paclitaxel
